# Concluding remarks: *Good* vibrations at interfaces as probed with spectroscopy

**DOI:** 10.1039/d6fd00105j

**Published:** 2026-06-11

**Authors:** Bert M. Weckhuysen

**Affiliations:** a Inorganic Chemistry and Catalysis, Institute for Sustainable and Circular Chemistry, Faculty of Science, Utrecht University Universiteitsweg 99 3584 CG Utrecht The Netherlands b.m.weckhuysen@uu.nl

## Abstract

The 2026 *Faraday Discussions* meeting on the topic of “Vibrations at interfaces” brought together a group of scientists to address the forefront topics and related challenges and limitations when using different forms of infrared and Raman spectroscopy to interrogate the surfaces of chemical systems from both life and materials sciences. The invited, oral and poster presentations at the meeting nicely illustrate the great progress made in the past years to (a) capture the chemical complexity under realistic conditions (*in vivo*, *in vitro*, *in situ* and *operando*), (b) investigate the surface *in silico* and (c) track the changes taking place in both space and time. The following text demonstrates these three areas by using examples from the field of catalysis. The first example discusses the use of photo-induced force microscopy to investigate the zeolitic–imidazolate framework surfaces while interacting with formaldehyde. The second example focuses on the use of infrared spectroscopy of the CO_2_ methanation reaction thereby focusing on how we can assign infrared features to surface species thereby making use of theoretical calculations. The last example discusses the use of time-gated Raman spectroscopy to track the catalytic cracking of *n*-hexane.

## Abstract 2

The “Vibrations at interfaces” *Faraday Discussions* meeting addressed the forefront topics and related challenges and limitations when using different forms of infrared and Raman spectroscopy to interrogate the surfaces of chemical systems.

## Graphical abstract

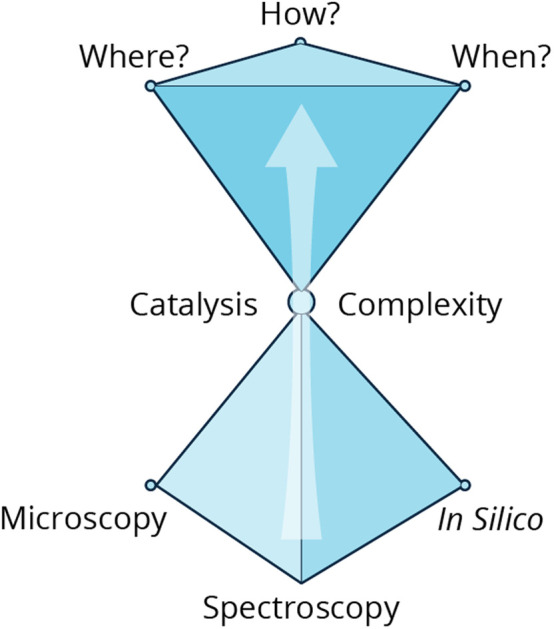

## Introduction

Vibrational spectroscopy, comprising infrared (IR) and Raman spectroscopy, is at the heart of analytical sciences.^1,2^ It is one of the most used methods in chemistry labs. This is due to (a) easy access and the relatively low price as many instrument manufacturers provide a wide range of equipment and (b) the relatively simple underlying basic principles, which are commonly taught in our university curricula. Furthermore, most IR and Raman spectroscopy instruments can easily be operated *via* computers, while vibrational data can be processed and analyzed with visually attractive software. This means that IR spectroscopy and to a lesser extent Raman spectroscopy are now important workhorses in modern laboratories in both academia and industry.

**Fig. 1:**
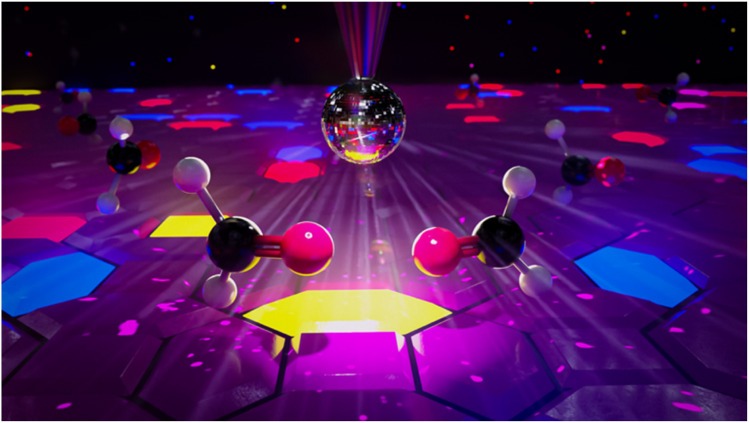
The theme of the *Faraday Discussions* meeting, held at Manchester Metropolitan University (Manchester, UK), focused on interfaces as probed with vibrational spectroscopy, thereby encompassing the wide range of capabilities of infrared (IR) and Raman spectroscopy. The conference title could also have been “*Good* Vibrations at Interfaces” as developments in this research field thrive on good collaborations within our community. The symposium brought together a diverse range of scientists, around 100 people in total, to discuss in an inspiring manner the latest advances of interfacial chemistry in the fields of both life and materials sciences. The image illustrates the sorption and conversion of formaldehyde at the surfaces of surface-anchored crystals of zeolite imidazolate frameworks. The interfacial chemistry has been probed with photo-induced force microscopy (PiFM), a more recently developed and explored nano-infrared microscopy methodology. After ref. 3.

The theme of the *Faraday Discussions* meeting – held in the Dalton Building of Manchester Metropolitan University (Manchester, UK) on May 8–10, 2026 – focused on the chemistry at interfaces as experimentally probed with various forms of IR and Raman spectroscopy, and either inspired or backed by theoretical calculations and predictions (Fig. 1). This is summarized in Table 1. The conference was organized around **three main topics of chemistry at interfaces**: chemical complexity, the surface *in silico* and tracking chemical changes in place and time.

**Table 1:** Overview of the different approaches and methods discussed during the *Faraday Discussions* meeting, held in Manchester (UK, May 8–10, 2026), organized according to the main topics of chemistry at interfaces

Methodologies	Modes	Specific methods and/or topics
Far-field vibrational spectroscopy methods	Infrared	Sum-frequency generation (SFG), photo-acoustic spectroscopy (PAS), and fast-scan and step-scan modes
	Raman	Surface-enhanced Raman spectroscopy (SERS), and time-gated Raman spectroscopy
Near-field vibrational spectroscopy methods	Infrared	Photo-induced force microscopy (PiFM), and atomic force microscopy-infrared spectroscopy
	Raman	Tip-enhanced Raman spectroscopy (TERS)
*In silico* methods	Infrared & Raman	Density functional theory (DFT), and machine-learned force fields, …

**Chemical complexity** can be best understood by the fact that vibrational spectroscopy can interrogate various objects ranging from materials sciences (*e.g.*, electrodes, batteries, fuel cells, photovoltaic devices and catalysts) to life sciences (*e.g.*, organs, tissues, cells and biomolecules). For each of these chemical objects the trend is to study them experimentally as well as *in silico* as close as possible to their real operational conditions. Within this context, it is important to refer to a recent Technical Report of the International Union for Pure and Applied Chemistry (IUPAC), which discusses various Latin terms for sample characterization in both materials and life sciences.^4^ These terms for sample characterization include a.o., *in vivo*, *in vitro*, *ex situ*, *in situ*, *in silico*, *post mortem*, and *(in) operando*. This nomenclature (evolution) illustrates the continuous development of analytical methods, including vibrational spectroscopy, to investigate complex chemical systems. It starts from the notion that the understanding and prediction of the behavior of any complex chemical system starts with **tracking it in real operation as function of both space and time**. Hence, impactful chemical science can only be obtained when we study real-life systems under as realistic and hence as relevant as possible measurement conditions with a.o., vibrational spectroscopy.

**Fig. 2:**
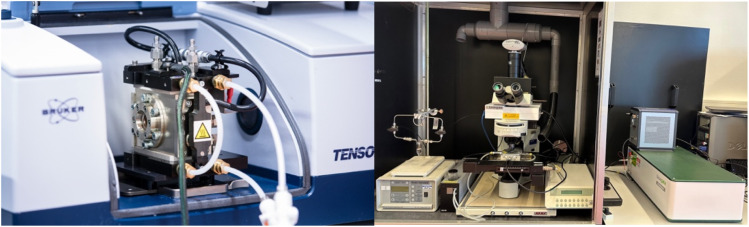
Pictures of *in situ* and *operando* spectroscopy set-ups for studying porous materials, while catalyzing chemical reactions available at Utrecht University. (left) Infrared (IR) spectroscopy setup used for carbon dioxide methanation over nickel-based catalysts; and (right) Raman spectroscopy setup used for catalytic cracking of hexane over zeolite-based catalysts.

This also holds for my own field of research: **heterogeneous catalysis**. Heterogeneous catalysis is one of the research fields where interfacial chemistry is at the heart of chemical conversion processes.^5–7^ More specifically, the catalyst surface provides the necessary interfaces with either a liquid or gas, where molecules collide, adsorb, react, desorb and ultimately leave. Vibrational spectroscopies are very suitable to probe the catalyst material, its surfaces, the surface reaction intermediates and spectator species, as well as the surrounding gas-phase and liquid-phase molecules. IR and Raman spectroscopy have their own sensitivity and specificity for providing bulk (of the solid, liquid-phase and gas-phase) or surface (at the liquid–solid and gas–solid interfaces) information. Hence, combinations of different vibrational methods are very powerful to shed insight into the molecular processes taking place at the interface of catalyst materials. Fig. 2 shows some pictures of *in situ* and *operando* IR and Raman spectroscopy equipment to track the complex interfacial chemistry of solid catalysts in action.

In what follows, I will outline my Concluding Remarks lecture at this *Faraday Discussions* meeting. More specifically, I highlight the above strategy using three showcases thereby focusing with three distinct vibrational techniques on the conference topics of (a) analysis of complex systems, (b) the surface *in silico*, and (c) tracking change. The first example discusses the use of photo-induced force microscopy (PiFM) to investigate the surface of zeolitic–imidazolate frameworks (ZIFs), namely ZIF-8, while interacting with formaldehyde.^3^ The second example focuses on the use of time-resolved IR spectroscopy of the CO_2_ methanation reaction thereby focusing on the formation of surface reaction intermediates and how we can assign the IR features to specific surface species thereby making use of DFT calculations.^8^ The system is a Ni/SiO_2_ catalyst. The last example discusses the use of time-resolved time-gated Raman spectroscopy to track the cracking of *n*-hexane over industrially used fluid catalytic cracking (FCC) particles.^9^

## Analysis of complex systems

Surfaces of porous catalysts are very heterogeneous both in their chemical composition and physicochemical structure. These heterogeneities may span many orders of magnitude, from the level of a single-atom and a single-molecule up to the level of millimeters of a single catalyst extrudate and the level of meters of a chemical reactor.^10–12^ In order to better appreciate these spatial heterogeneities, it is important to have the necessary micro-spectroscopy tools to investigate the complexity of catalyst materials.

A showcase from our group makes use of the technique of *in situ* PiFM, which combines atomic force microscopy (AFM) with IR spectroscopy.^3^ More specifically, *in situ* PiFM measurements in combination with DFT calculations allowed study of the sorption and conversion of formaldehyde into methanol and/or formic acid on the external surfaces of well-defined faceted ZIF-8 microcrystals with nanoscale resolution. Preferential adsorption of formaldehyde occurred on high index crystal planes, while *in situ* PiFM allowed visualization of unsaturated nanodomains within extended external crystal planes, showing enhanced sorption behavior of formaldehyde on the nanoscale.

**Fig. 3:**
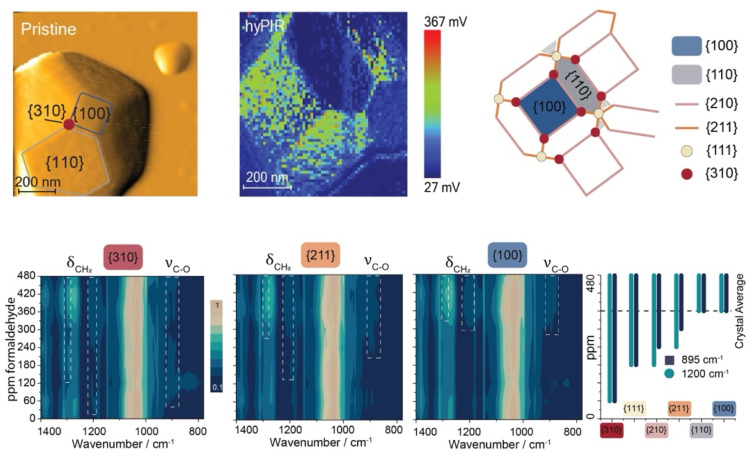
Experimental evidence of the structure-sensitive adsorption of formaldehyde on a ZIF-8 microcrystal. (top, left) Atomic force microscopy (AFM) image of a ZIF-8 crystal showing the expression of different crystal planes. (top, middle left) Hyperspectral images, with a full infrared (IR) spectrum at every pixel, during exposure to formaldehyde. (top, right) Overview of the different ZIF-8 facets, edges and corners. (bottom) Contour plots show the formaldehyde sorption-induced changes for a {310} corner (left), a {211} edge (middle, left), and a {100} facet (middle, right). (right) From these contour plots, formaldehyde response pressures, *i.e.*, the pressure at which an IR response was recorded, are presented. Reproduced with permission from ref. 3. ©2023 The Authors. *Nature Communications* published by Springer Nature.

Some illustrative AFM and PiFM data are shown in Fig. 3, thereby demonstrating the structure sensitive adsorption of formaldehyde on the surfaces of a ZIF-8 microcrystal.^3^Fig. 3 shows a set of hyperspectral images, with a full IR spectrum at every pixel, when a ZIF-8 microcrystal is exposed to formaldehyde. Adsorption of formaldehyde was observed through the increase in the IR bands at ∼1280, ∼1200, and ∼895 cm^−1^, corresponding to the *δ*_CH_2__, rock, *δ*_CH_2__, wag, and *ν*_C–O_, respectively. The *ν*_C=O_ vibration of physisorbed formaldehyde was not observed as the C=O double bond was broken due to strong chemisorption of formaldehyde on the ZIF-8 surface. It was found that ZIF-8 corners, edges and facets behave differently when exposed to an increasing amount of formaldehyde, thereby revealing that the reactivity increases in the order: facets < edges < corners.

## The surface *in silico*

Molecular structures at catalyst surfaces are clearly different from those of the bulk. As a consequence, we do not have (always) proper reference compounds and related vibrational fingerprints to benchmark experimental data. Theoretical calculations may be of help, but it is clear that these theoretical calculations are not yet at the required level to make spectroscopic fingerprinting of surface species unambiguous.

In a recent showcase from our group we have performed DFT calculations to determine the spectroscopic signatures of plausible surface species present on nickel metal nanoparticles supported on a silica support.^8^ In the past years, we have investigated Ni/SiO_2_ catalysts in detail as they are active in the CO_2_ methanation reaction. In an attempt to elucidate the reaction mechanism of CO_2_ methanation, we have performed *operando* IR spectroscopy measurements on differently sized SiO_2_-supported nickel metal nanoparticles (Fig. 2, left panel). By doing so, we have observed that the surface species are dependent on the nickel metal nanoparticle, and that the CO_2_ methanation is a structure sensitive reaction, at least for small nickel metal nanoparticles.

**Fig. 4:**
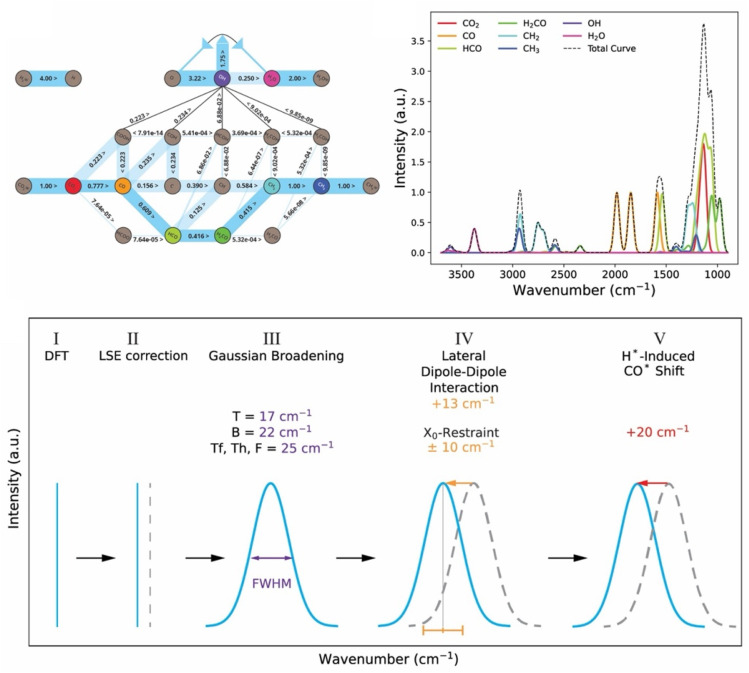
(top, left) Schematic overview of the flux diagram for CO_2_ methanation over a Ni(110) surface, which has been determined to be the most active surface in a Ni/SiO_2_ catalyst. The predominant reaction pathway is visualized with an opaque blue bar. The nodes representing the stable surface reaction intermediates along this pathway are color-coded. (top, right) Theoretical infrared (IR) spectrum constructed from the intermediates involved in the dominant reaction pathway for CO_2_ methanation over a Ni(110) surface. Vibrational frequencies were broadened using Gaussian functions with a Full Width at Half Maximum (FWHM) of 25 cm^−1^. The colors of the spectral contributions correspond to the node colors of the respective intermediates in the panel in the top, left. (bottom) Schematic overview of constructing an IR spectrum in the CO region. I. Fundamental CO* vibrational frequencies are computed using Density Functional Theory (DFT). II. Frequencies are corrected using a Linear Scaling Equation (LSE). III. Peak broadening—accounting for energy–time uncertainty—is simulated by applying a Gaussian function with site dependent FWHM: 17 cm^−1^ for top (T), 22 cm^−1^ for bridge (B), 25 cm^−1^ for threefold (Tf, Th) and fourfold (F) sites. IV. To approximate the effect of dipole–dipole interactions at ∼0.2 ML CO* coverage, peak positions are shifted by +13 cm^−1^. An uncertainty range of ±10 cm^−1^ is applied (X_0_-restraint). V. At 300 °C CO_2_ methanation conditions, with ∼0.55 ML H* coverage, CO* frequencies are further increased by +20 cm^−1^ to account for H*-induced CO* shifts. Reproduced with permission from ref. 8. ©2026 The Authors. *ChemistryEurope* published by Chemistry Europe and Wiley-VCH GmbH.

Fig. 4 shows a selection of the DFT results obtained for the most active surface, namely Ni(110), for which the most likely reaction pathway has been calculated (top, left image), including the potential surface reaction intermediates to be probed with vibrational spectroscopy.^8^ A theoretical IR spectrum constructed from the predicted surface intermediates involved in the dominant reaction pathway for CO_2_ methanation over Ni(110) is included in Fig. 4. This theoretical spectrum reveals the complexity of pinpointing surface intermediates as their spectroscopic fingerprints are (partially) overlapping, and it is (mostly) unknown what their relative surface occupancies as well as their spectral intensities are. This is already complex for the relatively simple CO* molecule adsorbed on a Ni surface, as shown in the bottom panel of Fig. 4. This figure shows the gradual construction of a theoretical spectrum in the CO region. In a first step, the fundamental surface CO vibrational frequencies are computed using DFT. Subsequently, these fundamental frequencies are corrected using a Linear Scaling Equation (LSE). In a third step, peak broadening, thereby accounting for energy–time uncertainty, is simulated by applying a Gaussian function with site dependent Full Width at Half Maximum (FWHM). To approximate the effect of dipole–dipole interactions, the IR peak positions have been shifted with a constant wavenumber. In a final step, the surface CO vibrational frequencies are further increased to account for H*-induced CO* shifts.

Hence, we have presented a method to correlate calculated vibrational frequencies with measured IR spectra for the CO_2_ methanation reaction over Ni surfaces. Fig. 4 highlights the reconstruction of the CO*-region, thereby demonstrating how this benchmark approach aids in IR peak assignment, but also reveals the inherent complexities and related limitations of interpreting *operando* IR spectra obtained during catalytic reactions.

## Tracking change

Vibrational methods are ideal to track physicochemical changes within solid materials during catalysis. Both IR and Raman spectroscopy have a time-resolution, which is relatively fast (*e.g.*, sub-seconds) thereby capable of studying changes taking place within the catalyst material and at its active surfaces. It is now generally appreciated that catalysts are very dynamic and change both in time and space. Furthermore, there are also substantial differences between catalyst particles (interparticle heterogeneities) and within one single catalyst particle (intraparticle heterogeneities).^10–12^ Tracking these changes and appreciating the associated heterogeneities have turned out to be essential in better understanding reaction and deactivation mechanisms in a wide variety of chemical processes.

A recent showcase from our research group makes use of the technique of *in situ* time-gated Raman spectroscopy (Fig. 2, right panel).^9^ More specifically, Raman and fluorescence (lifetime) micro-spectroscopies have been used to study the formation process of coke deposits of FCC materials during the cracking of hexane at the single-particle level. Excitation with a pulsed laser induces both Raman scattering and fluorescence and subsequently the collected signal has been split over two detectors, which allows recording of Raman spectra and fluorescence intensities and related lifetimes simultaneously. It was found that there were considerable interparticle heterogeneities as the degree of graphitization and the fluorescence lifetime *τ̄* vary greatly from catalyst-particle to catalyst-particle within a sample. Averaging 75 Raman spectroscopy measurements per sample clearly revealed a higher degree of graphitization for FCC materials more exposed to hexane. These trends have been confirmed with *in situ* time-gated Raman spectroscopy measurements, thereby offering insights in the relationship between the type of coke (precursor) molecules and lifetime *τ̄* values.

**Fig. 5:**
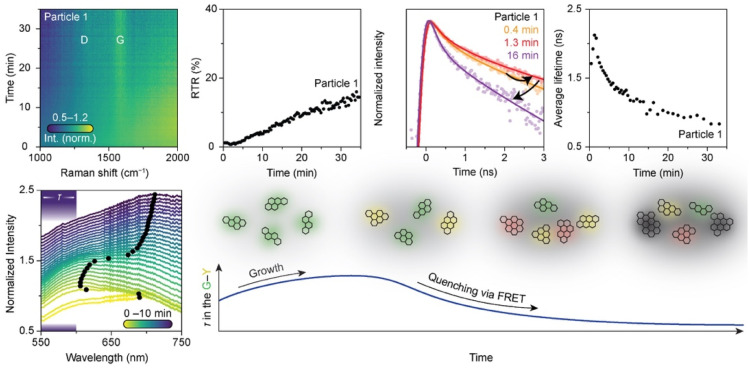
Simultaneous single-particle *in situ* Raman and lifetime micro-spectroscopy during hexane cracking over a fluid catalytic cracking (FCC) material. (top, left) Heatmap of the Raman spectra of FCC particle 1 recorded with the time-gated Raman spectrometer during hexane cracking. (top, central left) Raman-to-Total Ratio (RTR) as a function of reaction time. (top, central right) Normalized fluorescence decay traces recorded after 0.4, 1.3, and 16 min of hexane cracking. (top, right) Average fluorescence lifetime *τ̄* as a function of reaction time. (bottom, left) Fluorescence emission spectra of FCC particle 1. The black points indicate the maximum of the spectra. The grey area illustrates the wavelength range of the fluorescence lifetime measurement, corresponding to molecules emitting in the green and yellow spectral region. (bottom, right) Simplified representation of the formation and growth of poly-aromatic molecules on the surface of an FCC particle. It is important to note that the drawn molecular structures are not suggestions for the assignment of the data to specific molecules, but rather a suggestion for the trends in the growth of polyaromatic and graphitic structures. Reproduced with permission from ref. 9. ©2024, The Authors. *Angewandte Chemie International Edition* published by Wiley-VCH GmbH.

An example of a set of acquired *in situ* time-gated Raman spectroscopy data, thereby revealing the distinct D and G band intensities as a function of reaction time, the fluorescence intensity and *τ̄* changes as well as the related interpretation on the formation of coke deposits within FCC particles, is shown in Fig. 5. These data for a single FCC particle (labeled as 1) indicated the transformation of polyaromatic species into graphitic carbon, as illustrated by an increase in the Raman-to-Total Ratio (RTR) values. The small rise and subsequent decline of *τ̄* indicate a change in the type of coke (precursor) species. Moreover, these changing fluorescence dynamics, together with the fluorescence redshift indicate a prolonged presence of smaller coke deposit species of which the fluorescence is quenched more and more by adjacent larger poly-aromatics that can act as Förster resonance energy transfer (FRET) acceptor molecules.

## Conclusions and future prospects

The *Faraday Discussions* meeting on “Vibrations at interfaces” brought together experts with different expertise from all over the world. We all shared our passion to push the technical boundaries of current and emerging vibrational spectroscopy methods to investigate complex chemical systems, ranging from materials to life sciences. It became also clear that there is an increasingly powerful set of experimental and computational techniques available to investigate and more realistically track the complexity of interfacial chemistry at the different time and length scales of importance.

**Fig. 6:**
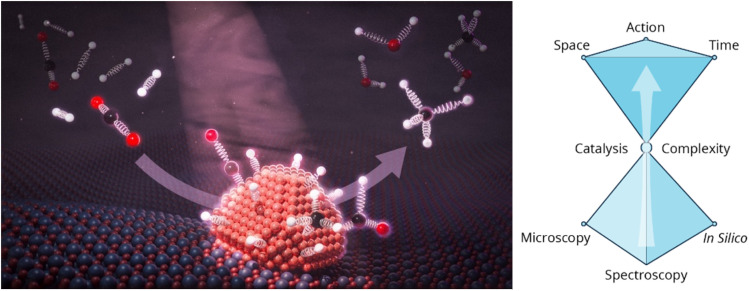
(left) Snapshot of a molecular movie, as probed by *operando* vibrational spectroscopy and backed by advanced theoretical calculations, for a single metal nanoparticle converting CO_2_ and H_2_ into CH_4_ and H_2_O. How far are we in developing the experimental and theoretical tools to make such a movie thereby visualizing what is happening at the level of a single-particle and single-molecules either in the gas phase or adsorbed on the metal nanoparticle? Can we probe the chemical bond elongation towards breaking and the subsequent chemical bond making thereby tracking the individual reaction steps of a chemical process as function of both temperature and pressure? (right) Schematic capturing the future directions thereby answering the where (space), when (time) and how (action) of the complexity of catalytic processes by using in a truly concerted manner microscopy, spectroscopy and *in silico* approaches.

As also illustrated by the three showcases above, I am convinced that we did not yet reach the technical limitations of IR and Raman spectroscopy. Certainly, much more can be learned in terms of pushing the spatial and temporal resolution to track the complexity of chemical systems, as well as to more accurately describe the surface *in silico* by using *ab initio* calculations and artificial intelligence tools. Computational methods will become increasingly predictive thereby moving away from model chemical systems to more realistic chemical systems. Similarly, experimental methods are moving away from *ex situ* and *in situ* towards true *operando* as the behavior of a chemical system lies in its operation itself. This is fully evident for catalytic processes. The envisaged end result is the capability to make *operando* molecular movies, as illustrated in Fig. 6 (left). Ultimately experimental and computational methods have to be used in a truly concerted fashion thereby not only explain, but also predict the behavior of chemical systems, such as catalytic processes (Fig. 6, right).

An intriguing question, which was also central to the conference, is: “What is the scientific question, and what is the best vibrational spectroscopy technique to answer this question?” It brings us to an apparent paradox in analytical sciences. On one hand, there is an ever-growing list of vibrational spectroscopy methods at our disposal, and by using them we learn more about chemistry. This leads to the formulation of new scientific questions. On the other hand, by harvesting new knowledge about complex chemical systems we suddenly realize the limitations of existing vibrational spectroscopy methods. This humble appreciation leads us to realize that we as a scientific community have to push strongly vibrational spectroscopy methods to the next level.

I am convinced that this *Faraday Discussions* meeting made all attendees realize the power and limitations of currently used experimental and theoretical methods, and to ask what’s the next big thing in vibrational spectroscopy and microscopy. The future will tell which directions our scientific community have been taking.

## Conflicts of interest

I have no conflicts to declare.

## Supplementary Material

FD-265-D6FD00105J-s001

## Data Availability

No primary research results, software or code have been included and no new data were generated or analysed as part of this perspective article. Supplementary information (SI) is available. See DOI: https://doi.org/10.1039/d6fd00105j.
